# Hindu Response to Dying and Death in the Time of COVID-19

**DOI:** 10.3389/fpsyg.2021.636384

**Published:** 2021-02-12

**Authors:** Purushottama Bilimoria

**Affiliations:** ^1^School of Historical and Philosophical Studies, University of Melbourne, Parkville, VIC, Australia; ^2^Indian Philosophy Lab, RUND University, Moscow, Russia

**Keywords:** COVID- 19, Hinduism, death, wisdom, India, empathy

## Abstract

We wake each morning to news on the glaring statistics of people infected by COVID-19 and others reportedly dying from complications thereto; the numbers are not receding in at least a number of countries across the world (barring a few that imposed strict lockdowns, testing and quarantining measures, such as Australia, Singapore, New Zealand and Vietnam). It is hard to imagine a moment such as this that most of us have lived through in our life-time; but it is a reality and public challenge that we can neither ignore nor look away from. In what follows I will explore perspectives on death from the Hindu tradition and the kinds of response—and solace or wisdom—afforded by the tradition to the *angst* and fears evoked by this pandemic situation. In concluding the discussion, I shall offer tentative reflections on how the Hindu perspective may be universalized, such as might invite conversations with therapists and care workers who may be seeking alternative resources to help expand the therapeutic space in more beneficent ways during the Covid-19 pandemic and its after-effects

## COVID-19 Epidemiological Data

WHO reports in the closing week of January 2021 that there have been some 1 billion and 500,000 confirmed cases of COVID-19 infections across the globe, half of which have been in the Americas (4.4 million, with close to 25.2 million alone in the United States); Europe has had 33.5 million cases and South-East Asia 12.7 million (of which India has had 10.7 million cases, with a rapid downward curve); Brazil 9 million. While the global acceleration in case incidence has slowed down – around half-a-million new cases reported on average every 24 h- death rates, however, continue to increase with over 100,000 new deaths reported in 2021, bringing the global total to 2.2 million; the United States (over 421,600), South and South-East Asia (200,000) and extended Europe (730,000, largest single cluster in the UK), remain the lead contributors to death through COVID-19; and it is escalating egregiously in some parts of the globe^1^. The world is experiencing a tryst with destiny and its people are living through difficult times which calls for a phenomenological response to this new threat of unnatural and in the majority of cases untimely death.

## Hinduism and Death

Since the large percentage of those infected and dying from COVID-19 in South Asia (and the diaspora), are Hindus it behooves me to focus on the Hindu perspective on dying, death, and dealing with the traumas associated with the challenges of mortality. We shall ask toward the end of the discussion if the Hindu response could have fruitful application in the context of the wider challenges of the COVID-19 pandemic.

We begin with the question, whether in Hindu thinking a distinction is made between the body and the soul; and if so what be fate of the body and the destiny of the soul? The closet cognate in Sanskrit to “soul”(*psychē)* is “ā*tman*” which refers to the subjective unity of the self-consciousness of the individual and captures their vital and cognitive or conscious and unconscious functions. The combination of the vital, cognitive and conative functions is better known as the indwelling *jīva*, which is often combined with “ā*tman*” to read as “*jīva-ātmā*” *(“*spirit,” “self,” or psychical self), since the ā*tman* always remains the timeless essence of the individual, regardless. The latter is a useful ontological signifier for that which dwells within the individual, provides the life-breath (*prāṇa)*, mental prowess, and volition, and which ultimately departs at death; the transcendental self (or Self) that is denoted by ā*tman* has the secondary connotation of being a living agency much as one would speak of the “soul.” At face value, there might seem to be a dualistic relation between the *jīva-ātmā* and the body; however, this is not to be understood in Cartesian terms—i.e., complete separation of “spirit-mind” and “body,” the corporeal matter—but rather as a more intertwined and interdependent relation or the triunity of “spirit-mind-body”; so it is a distinction without difference, and certainly not a separation, perhaps not until the point of death (Bilimoria, 2016).

So what happens at death? The first point to note is that death remains an inevitable condition of sentient existence. Whatever has birth, grows through nutrients, heat, time, air, ether (space) and breath; but it also faces decay of the elements making up the fractious body that leads to death in its time-cycled existence.

At the point of death, the *jīva* is believed to leave the body, escorted by Yama (Lord of Death) who, much like Thanatos, controls the destiny of the individual beyond the earthly realm. While departing, the *jīva* takes with it as its propulsion force *prāṇa*, or “vital breath” conceived non-corporeally—akin to Bergson's *elan vital*—as the underlying principle of sentience. This occurs as soon as the “trace-data” curled from the memory bank and subliminal psychical dispositions have been uploaded; and since the mind of the individual is believed to be “extended,” the process may continue outside of the deceased body. Death is indeed of the body (the terminal cessation of heart, organs, and brain), though not of the spirit-mind. This indicates that the spirit-mind while the individual was alive had been fully integrated and embedded throughout the biological body-frame.

Once the “uploading” is complete and the mind is freed from the shackles of the mortal remains, the shepherding *jīva* is said to move through various dimensions in rapid succession: the *jīva* might sift through light-scented tunnels, or spiral through energy-fields suffuse with welcoming god-like beings (*devas*). Some NDE-reports suggest sighting of ancestors and teachers as well, with garlands, fanfare and celestial music, or frighteningly disturbing clamor in other cases, as the *jīva* is whisked away to some celestial location. However, the connection with the body back on earth and the surrounds or family is not completely severed for another three or so days. In India it is normative for Hindus to cremate the corpse within 24 h. The underlying belief here is that the *jīva* of the departed is relieved from the “higher planes” to be present at the last rites, both out of attachment to the prior body but also to bear witness to the completion of their mission on earth and cherishing in spirit-state the last moments with family and well-wishers (Mishra, 2010).

But if it is believed that the “soul” as ā*tman* is timeless, might there be a peaceful resting place, such as heaven as we have in certain of the world's religions? Indeed, as the seminal *Bhagavad Gītā* (BhG, 1981) states, *jīva*'s existential core, i.e., the ā*tman*, never dies, neither is it born, nor can it be cut, burnt by fire, moistened by water, weltered by wind, or overrun by time, because it is eternally immortal and pure: death cannot come anywhere near the ā*tman*. Knowing it as such, there is no cause for lamenting (2.23–25).

The scriptural accounts, however, differ as to where exactly the surviving *jīva* ends up after it after leaving the earthly body: in Krishna's universal body or his heavenly abode, or in another body, or finds liberation from the cycle of birth and birth; or sequentially each of the above? One thing nevertheless is clear that the after-life is not conceived in *linear temporal* terms, but rather in cyclical or curving time-dimensions that far exceed the four dimensions of the natural world that we inhabit.

The philosophical Upaniṣads (2008) speak of two alternative destinies depending on the person's stock of *karma* (action-traces) mitigated by the effective performance of one's *dharma* (right duties and normative practices) in the life just concluded (perhaps compounded with as-yet unresolved karma carried over from previous existences). If a person has performed three requisite rites in the fire-altar and departs during the sun's northern journey and in moonlight, they reach *Brahma- or devaloka* (the realm of the supreme deity), from which there is no returning; if one departs during the “night of the smoke” or long winter solstice and dark-moon, then *pitṛaloka* (hierarchical dwellings of the foreparents), is reached, from which one returns to another life-mode.

There are nevertheless variations in certain of the Upaniṣadic texts, couple of which are worthy of noting. Release of the soul from the conditioned life on earth may lead to immortality in the form of eternal state of bliss *(ānanda*), most likely in an astral or spirit-form embodiment, marked by complete fulfillment of all desires and cravings and absence of pain and pleasure. Another account suggests total oneness with the Supremely Divine Effulgence *(paratparaṃ puruṣa divyam)*; yet another underscores indistinguishable merger with the Absolute as Brahman. They also speak of retributive hellish realms for those who may have committed excessive felony, trespassed *dharma's* rites, or violated rights of others. Where one ends up in the postmortem condition is determined by the autonomous clockwork of karma.

Each of the worlds yonder has its own path not of voluntary choosing, although there can be transitional movement from one world to the other, which is distinct from the cyclical journey of the *jīva* from one incarnation to the next in this world, or from death to death. After a period of reparation, the *jīva* undergoes metempsychosis and is reborn in another body with the past trace-memories intact in the unconscious, which puts a limit on their free-will.

So now faced with the fear death – in normal life and, especially, in the current pandemic, what kind of solace does Hinduism have to offer? Death, in Hindu thought, is indeed acknowledged to be a great mystery, an unknown; and yet one could arm oneself to avert attendant fears and *angst*. Fearing death is not considered to be unnatural; the fear of death in one life might be exacerbated by cumulative fears of death from the countless past lives that each *jīva* has passed through. The classical text of Yoga (*Yoga-sutras*, YS, 1990) opines that fear of death to life is one of the five unwholesome mental states which have to be overcome by attaining enlightenment (YS 2.3). Fear of death and clinging to life—which is a form of attachment—constitute an affliction that bedevils everyone, both the wise and the motley ignorant (YS 2.9). Krishna consoles the despondent warrior, Arjuna, with a surprise epiphany proclaiming, “I am now become Death [Time grown old], the destroyer of the world;. you should have no fears” (BhG 11.32).

The broader tradition itself suggests various means through which one could come to accept impending death, even advancing its adjudged inevitability, rather than prolonging life. The solace is more in way of being reminded that death is not the end of life, but rather that, as Mahatma Gandhi put it, “life and death are phases of the same phenomenon, the reverse and observe of the same coin.; life becomes livable, only to the extent that death is not treated as an enemy but as a friend…. death is not a fiend, it is the truest of friends. It delivers us from agony.” (Mahatma, 1960, 3, 4; 2, 237). Again, very often the sagely sermon of Krishna is recited to provide some succor to persons – especially in terminal state or who have been overcome with excessive *angst* about death as a possibility or as a reality facing them: “As a person casts off worn-out garments and puts on new ones, so the embodied *jīva* casts off the worn-out body and enters other new ones.” (BhG 2.22). Our thrownness onto death—to borrow an adage attributed to Heidegger—is not something to be avoided but rather to be countenanced and faced with courage and boldness, as does a martyr upon their calling to duty of justice. Furthermore, the *Yoga-sutras* and a number of other yoga texts prescribe various yogic (or spiritual) practices that help the adept to overcome “fear, agony, despair, helplessness and other feelings [that are] embedded in one's being” and which pertain as much toward death (YS 4.11). The Bhagavadgītā follows up with a proposition that if one could cultivate the alternative emotion of detachment (*asakti*) as a virtue in its own right (3.25), freeing oneself from the temptations of desire to go on existing—as if immortality was the order of the universe—and its counterfactuals of anger, fear and loss of hope, then one would achieve a state of reasonable intelligence (*vyavasāyātmikā buddhir ekeha*, 2.41). In this resolute state one determines the best course of practical action grounded in steadfast disposition. This is a normative heuristic, not a categorical imperative, for virtues no more than emotions can be prescribed; they can only be cultivated, prophylactically and pedagogically in a cultural setting. Armed with this rational wisdom, as in a battlefield, one confronts the menace of death.

The next question that follows from the foregoing is: how might one prepare for death? Does Hinduism provide directives those afflicted and nearing death to face steadfastly their dying moments? The sage advice is that what we ought to do in life is to work toward attaining a state of selfless awareness or a preparatory spiritual disposition “of steadfast constitution” (*stithaprajña)* that would unburden the ubiquitous fear of dying when the fatal moment arrives. And then there would be no need for mourning, or grief either in the aftermath, on the part of those living and left behind, who we call the surviving kin and associates. The trauma of dying need not be debilitating; it could indeed be transformative: there is indeed hope, and hoping.

A Hindu person will spend a good part of their life preparing for a new life which may or may not entail re-embodiment in a physical body in the after-life; they may intentionally wish for an eternal life in the *devaloka*, should they be so eligible. But there may be number of normative practices that might well prepare one for death. These could range from virtuous excellences—such as the courage and temerity to face any untoward consequence, however fearful and threatening to life these may be—to more concrete praxis such as regular purposeful fasting that would both cleanse the body of toxins and sediments from medications, and help ground one in the suffering that the body might recurrently experience when deprived of food—as may be the predicament at the point of death and in the aftermath, for that is an unknown. The cultivation of this disposition is reinforced with constant practice of contemplation, meditation, devotion or supplication to the gods and ancestors with mantra-recitations, and counseling of a guru or care-therapist. The profess intent here is to seek release from mortality and fear of death—often personified, again, in the image of Yama, or indeed of Kāli, the ferocious feminine deity who adorns herself with a garland of skulls—a stark reminder of death—trembling a bloodied sword in one hand and a severed head in the other. One is also expected to have fulfilled all duties and obligations while in the embodied community, and settled all debts in the transactional world of commence and good-willed interrelations.

There really is no short-path to what might be called “the yoga of dying.” Likewise, again, the *Bhagavad Gītā* ordains obligations, sacrifice, askesis and other ascetic disciplines that are intended to free one from inclinations and impulses that chain us to the relentless wheel of birth and death, and have their intent set on *mokṣa*, or salvific liberation. These disciplines too are variously called “*yoga*” by Krishna (BhG 17.15–25). To aid the departing *jīva* in its onward journey to the appropriate or karmically-assigned destiny in the beyond, there is emphasis placed on proper demeanor and certain mandatory rites, observances and ritual preparations in the period before the last breath is taken, and after the life-breath has left, i.e., the work of death (*yama)* is completed. In the Tantric (esoteric) tradition, contemplative visualization of one's death is practiced, often in cremation grounds; even a concluding yoga-posture emulates the corpse-state (*shavasana*). We may also note the particular practices and ceremonies associated with cremation—or burial, depending on the status of the person—and after, and their continuing importance in the Hindu life-world. The mystic power of mantras (syllabic sound-forms) recited during the service, with other Vedic (prescribed scriptural) offerings (ghee and rice grains, sandalwood) to the god of fire (Agni) upon lighting the funeral pyre, are powerful moments meant both for a smooth apparitional transit of deceased (i.e., the *jīva*) to the beyond, as much as for the mourning community left behind on the ground: the two seemingly estranged parties might even stand together momentarily in an uncanny transcendental unity (or *communitas*) (Sherma and Bilimoria, 2020, p. 94, 163, 170, 200).

Finally, would one say that there is room for a “good and dignified death”? Certainly, the practices and rituals at the time of dying are of particular importance toward cultivating an equanimous state that would have the challenged individual prepared for an unperturbed arrival of death. A resolutely peaceful death where one has the sense of having fulfilled life's mission and completed all that was expected by the normative order—presumably the gods too or the larger tradition (*dharma)*—accounts for what could be called a “good and dignified death.” Without an antecedent good-life one cannot expect a consequent good-death; it is not simply a matter of grace or forgiveness from some divine supremo. There might be room for remorse for the wrongs one might have done, the injuries caused to others, and obstacles one might have put in the path of the societal or greater *dharma* running its course, that may mitigate the severity of the post-mortem suffering; but there will be no grief or mourning for one's own death (here or in the hereafter)^2^.

This motif is brought out rather colorfully and movingly in the retelling of the concluding hours of the battle between two warring fraternal clans in the classic epic, *The Mahābhārata* (MBh, 2009), by the poet Bhāsa (3rd CE) where Duryodhana, the scion of the so-depicted “bad guys,” is struck down in the bitter internecine battle by an opposing warrior. Even though the manner of the defeat was in violation of the rules of war, he was nevertheless overcome with remorse precisely for reasons of the excesses of his own insatiable ambition (to lay claim to the father's kingdom), as well as for the deceits, war-mongering trajectory, and the horrendous evils he had trumped up as the elder leader among his brothers upon the cousins who were favored by the aging and infirm king as the rightful heirs.

Bleeding profusely from the insufferable blow of Bhima's mace around his thighs, Duryodhana declares that he is dying but that this is to be a “glorious death”; in many words, he tells his bereft parent and his son who are overwhelmed with grief that they should not be concerned because the warrior-hero will soon join his hundred brothers/cousins (among those also who his forces have killed), indeed that he is “reborn today,” freed of the malice he had long harbored for his fraternal counterparts, and instead is overcome with magnanimity and compassion for their eldest, Yudhisthira, leader on the competing side. His conscience now reminds him in fast-moving images of the wicked and malevolent acts and decadent intentions he had indulged himself in the tussle for power, directed at the opposing filial community; but he also feels fulfilled in his heart as he witnesses his own son being coronated by his Guru's son. He continues: “My life is now slipping away. I see my ancestors, my hundred brothers, and Karṇa [protégé brother] ahead of them; I see Abhimanyu (youngster from the rival side who has killed in a staged ambush), seated on [god] Indra's elephant rebuking me. Urvaśi and other heavenly nymphs have come to welcome me; here are the oceans to greet me. Lo, the river Gańgā too is greeting me with love. Yama with chariot drawn by thousands of swans has come for me. Here I go to meet them all.” With these words, Duryodhana breathed his last. This in the tradition is what would be considered a “good and dignified death.”

## Discussion

It is believed in the Hindu tradition that death, detachment and grief are not unrelated psycho-tropes of human experience. When the imminent death of a dear or near one, or in someone's care, calls another or others to grieve and mourn, they should have the courage, faith, love and support to remain open to the pain and the loss in the situation, and introjectively exude empathy (Bilimoria, 2012; Zeeshan et al., 2020).

The keen reader however may still wonder whether the responses and pathways discerned from certain classical texts and cultural moorings remain confined to beliefs and praxis of caste-based Hindus as a whole; and the corollary: whether these bear any relevance outside of his cultural context to clinicians, therapists and care providers more widely who are confronted with the intense suffering and/or the dying moments of patients afflicted with COVID-19, or other fatal conditions, but who may come from different faiths or no-faith at all and in very different cultural location (i.e., they belong to the largely secular modern world)? At the risk of seeming to excessively decontextualize (some would say demythologize) a historically and culturally rooted psycho-religious normativity, I would venture to suggest—following the 7th−8th century (CE) doyen of Indian philosophy, Ādi Saṅkara—that the central teaching of Hindu ethics and eschatology, especially that articulated in the *Bhagavadgītā*, are amenable to universalization—just as Gandhi transformed the localized Jaina moral vow of non-injury (to microbials and all creatures) into a forceful principle of nonviolence that found broad application in civil, political and ecological spheres as well.

A therapist therefore may improve their practice by looking through the broad lens of Hinduism. This will not only help develop a deeper understanding of a client's cultural and religious beliefs but also provide concrete examplery case-points to present to the broader class of patients. For instance, the therapist may offer her client alternatives to their preconceived “interpretation of death,” regardless of whether the client is religious, agnostic, non-theistic, or an atheist. These examples can provide the therapist with a more expansive toolkit to draw from and utilize in the therapy sessions. Hindu (along with Buddhist) philosophy and theology have already brought more widely through various channels, media and scientific experimentations a rich repertoire of ideas, teachings and practices that are now interactively part of the mainstream culture and vocabulary in our modern, secular, globalizing world: these include the well-known ideas about the law of *karma* and wheel of *samsara*, but also the much popularized practices of Yoga, muscle relaxation, meditation (mindfulness, transcendental attention, vipassana, walking, silent), mantra-contemplation, visualization, Guru-guidance, well-being retreats, puja-chanting, deity-devotion, affirmations, non-injurious (veggian) diet, Ayurveda-herbal treatments (with homeopathy and Himalayan-oil massage), tantra-chakra-kinesiology (akin to acupuncture) etc. A selection of these have rendered positive contribution to outcomes in, for instance, supporting cancer, PTSD, distressed-mental state, and palliative care, as well as toward wellness, psychotherapeutic and cognitive-aesthetic initiatives (often, understandably, stripped of the religious trappings of their Dharma-dhamma origins).

When a person with COVID-19 is stuck in a specific concept of death and has not achieved adequate psychological relief from it they may find strength in aligning their own psychic battles with Arjuna's struggles. Just as a client may believe their prayers are no longer being heard but then discover that daily affirmations or regular visualization practices bring about a profound sense of relaxation in their being and allaying of the experience of physical and emotional pain.

Hence there is compelling reason to believe that the Hindu position on death and dying opens up new conversation with therapists and care workers who may wish to expand their therapeutic space even as they deal with incidents and the after-effects of the Covid-19 pandemic. If for nothing-else then as a heuristic supplement to the normative framework and resources provided by current allopathic or clinical psychology praxis. These insights may make for the kind of solace that the philosopher Schopenhauer found on his death-cot as he heard passages recited from the *Upanisads*, or when Oppenheimer of the Manhattan (atomic-explosion) Project resorted to the verses in the *Bhagavadgītā* to describe the splendor of “thousand suns bursting forth,” while remaining opposed the development of nuclear H-Bomb for use in warfare. But beyond providing mere solace the potential of empowering those impacted to normalize their fears and welcome the supplemental strategies from Hinduism and other faiths toward the healing process need to be taken on board. We are here arguing and pleading for a paradigm shift in the discursive and practical approaches to the COVID-19 pandemic.

## Data Availability Statement

The original contributions presented in the study are included in the article/supplementary material, further inquiries can be directed to the corresponding author/s.

## Author Contributions

The author confirms being the sole contributor of this work and has approved it for publication.

## Conflict of Interest

The author declares that the research was conducted in the absence of any commercial or financial relationships that could be construed as a potential conflict of interest.

## Footnotes

^1^Available online at: https://covid19.who.int/ (accessed January 29, 2021).

^2^Of course, mourning and grief and a deep sense of helplessness or hopelessness on the part of the close kith and kin witnessing the dying and eventual passing of the loved one cannot be avoided. Space does not allow further discussion of this facet of Thanatos, which has been treated in much detail elsewhere, see Bilimoria (2012); in that paper I engage with some currents from western philosophical thinking on the more troubling or “heavy” passions and feelings. people everywhere experience grief and mourning; however, the emotions and feelings through which they express this intense state varies across cultures and possibly also history. I draw on a personal encounter with the death of dear ones that a colleague has narrated from their work as a care-therapist (Kaetz, 2020), and my own, that has led me to question and even overturn the neat theorizing and methodology afforded in Western studies on the emotions that are unleashed in such experiences. I turn instead to cross-cultural studies where “reciprocity” has a stronger presence than anything like a Kantian sense of duty—or “virtue ethics,” propositional judgment, the natural law imperative—of reconciling with forces higher or now-other than the mortals. Rather, I find a more perspicuous direction in psychoanalysis tempered with Indian phenomenology on the work of grief and mourning (see Sharma, 2014, p. 84–87).
